# miRNA-mediated risk for schizophrenia in 22q11.2 deletion syndrome

**DOI:** 10.3389/fgene.2012.00291

**Published:** 2012-12-13

**Authors:** Linda M. Brzustowicz, Anne S. Bassett

**Affiliations:** ^1^Department of Genetics, Rutgers UniversityPiscataway, NJ, USA; ^2^Human Genetics Institute of New Jersey, Rutgers UniversityPiscataway, NJ, USA; ^3^Clinical Genetics Research Program, Centre for Addiction and Mental HealthToronto, ON, Canada; ^4^Department of Psychiatry, University of TorontoToronto, ON, Canada; ^5^Department of Psychiatry and Division of Cardiology, Department of Medicine, University Health NetworkToronto, ON, Canada

**Keywords:** schizophrenia, genetic, miRNA, DGCR8, copy number variation

## Abstract

In humans, the most common genomic disorder is a hemizygous deletion of a 1.5–3 Mb region of chromosome 22q11.2. The resultant 22q11.2 deletion syndrome (22q11.2DS) can affect multiple organ systems, and most notably includes cardiac, craniofacial, and neurodevelopmental defects. Individuals with 22q11.2DS have a 20–25-fold risk of developing schizophrenia compared to individuals from the general population, making 22q11.2DS the strongest known molecular genetic risk factor for schizophrenia. Although the deleted region includes *DGCR8*, a gene coding for a miRNA processing protein, the exact mechanism by which this deletion increases risk is unknown. Importantly, several lines of evidence suggest that miRNAs may modulate risk for schizophrenia in other, non-22q11.2DS populations. Here we present a theory which mechanistically explains the link between 22q11.2DS, miRNAs, and schizophrenia risk. We outline the testable predictions generated by this theory and present preliminary data in support of our model. Further experimental validation of this model could provide important insights into the etiology of both 22q11.2DS and more common forms of schizophrenia.

## Background

Microdeletion of chromosome 22q11.2 or 22q11.2 deletion syndrome (22q11.2DS) (MIM #188400/#192430) is the most common human deletion syndrome with an estimated prevalence of 1 in 4000 live births (Goodship et al., 1998). Formerly known as DiGeorge or velocardiofacial syndromes, the expression is variable in severity and number of associated features (Bassett et al., 2011). While classically these include developmental features such as congenital cardiac and palatal anomalies, an often subtle facial phenotype and developmental delays and/or learning difficulties, later onset conditions are also commonly associated. These include hypocalcemia and thyroid abnormalities as well as psychiatric illnesses (Bassett et al., 2011). Over 60% of patients develop treatable psychiatric disorders by adulthood (Fung et al., 2010). In particular, about one in every four adults develops schizophrenia (Fung et al., 2010) and due to this high prevalence in 22q11.2DS patients, the 22q11.2 region is considered to be one of the main schizophrenia susceptibility loci in humans (Bassett and Chow, 2008; Insel, 2010).

**Figure 1:**
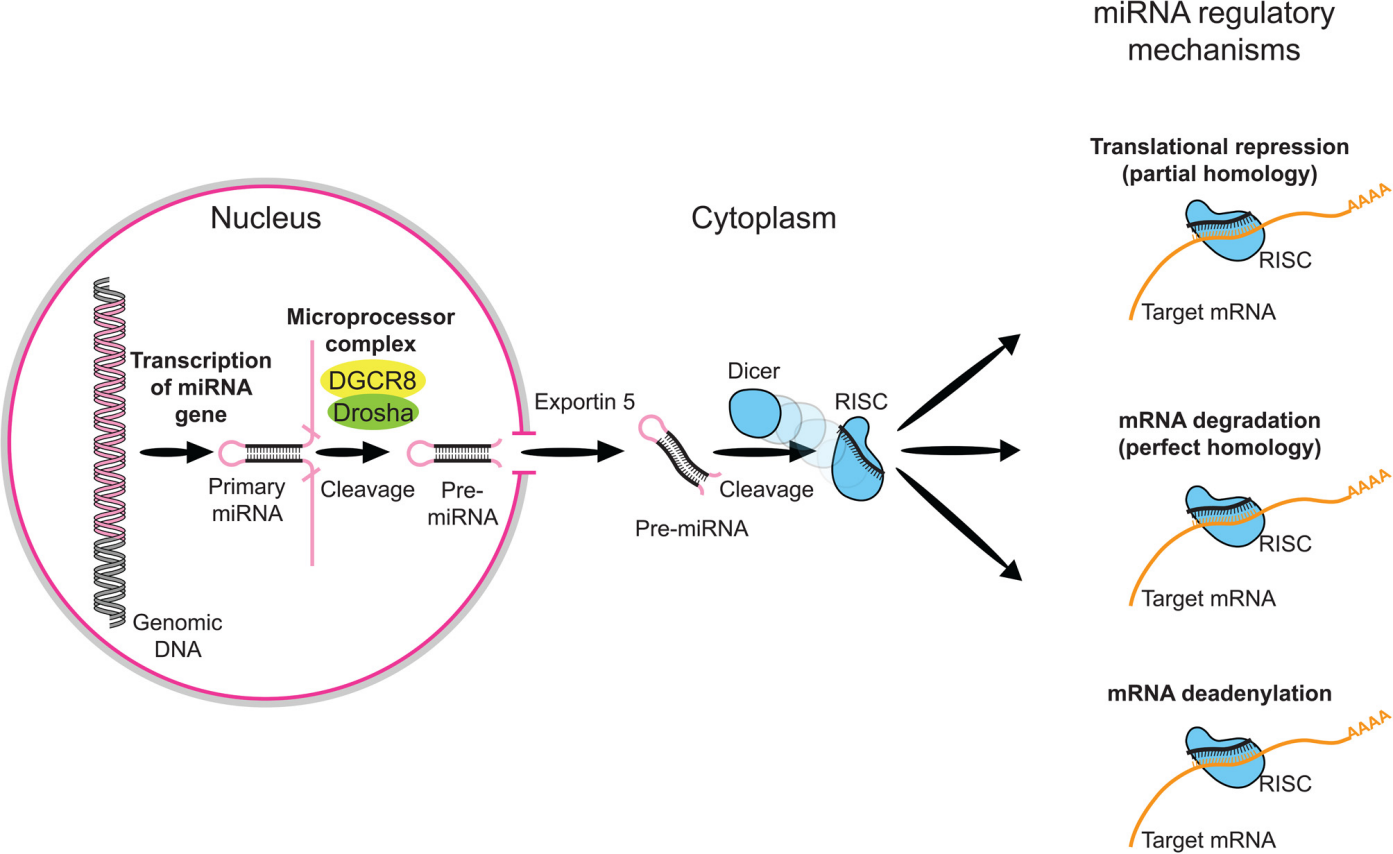
**Overview of miRNA processing.** MicroRNAs (miRNAs) comprise a growing class of endogenous molecules that regulate gene expression post-transcriptionally. By binding to partially complementary regions at the 3' end of messenger RNAs, these approximately 22-nucleotide single-stranded molecules direct target site recognition by the macromolecular RNA-induced silencing complex (RISC). RISC induces cleavage or translational repression of targeted transcripts. Mammalian miRNA biosynthesis begins with RNA polymerase II-dependent transcription, which yields long primary miRNA (pri-miRNA) transcripts. The pri-miRNA is processed in the nucleus to a pre-miRNA by the Microprocesser complex, which consists of Drosha, a member of ribonuclease III family (RNaseIII), and DGCR8 (also known as Pasha), which contains two double-stranded RNA binding domains and is responsible for pri-miRNA recognition. The pre-miRNA is then exported to the cytoplasm by Exportin-5 and further processed into a miRNA duplex by Dicer, another RNase III. One strand of the miRNA duplex is then incorporated into the RISC.

The 22q11.2 region is flanked by low copy repeats (LCRs) with greater than 97% DNA sequence identity. As for other genomic disorders (Kumar, 2008), the main mechanism giving rise to the hemizygous 22q11.2 deletion involves non-allelic homologous recombination that results from misalignment of LCRs during meiosis (Edelmann et al., 1999). Up to 90% of recombination events give rise to the common 3 Mb deletion (Shaikh et al., 2000), with approximately 8% of patients having a 1.5 Mb deletion, nested within the proximal region of the 3 Mb deletion. Rare smaller or atypical deletions have been reported but there is no evidence for specific genotype–phenotype correlations (Bassett et al., 2011). Over 90% of cases are found to have a *de novo* 22q11.2 deletion with the remainder found to be inherited from a parent. The commonly deleted region in 22q11.2DS encompasses approximately 45 genes and the consequences of decreased gene dosage of multiple genes are believed to be involved in phenotypic expression (Meechan et al., 2011). The molecular substrates underlying the different clinical features of 22q11.2 deletion, however, are still debated. One gene of particular interest, in the proximal deletion region, is DiGeorge Critical Region Gene 8 (*DGCR8*), which plays a critical role in the biogenesis of miRNAs (Figure 1).

Multiple lines of evidence have implicated miRNAs in general aspects of neurodevelopment, neurodegeneration, and brain function and specifically in schizophrenia (reviewed in Beveridge and Cairns, 2012; Mellios and Sur, 2012; Saito and Saito, 2012). A very large genome-wide association study of over 50,000 individuals identified a SNP in miR-137 as strongly (*p* = 1.6 × 10^−11^) associated with schizophrenia (Ripke et al., 2011). In addition, five genes associated with schizophrenia (*ZNF804A, CACNA1C, TCF4, CSMD1*, and *C10orf26*) have all been validated as targets of miR-137 (Kwon et al., 2011; Kim et al., 2012), suggesting that genes involved in schizophrenia susceptibility can be affected by either *cis*-acting mutations at the gene or via alterations in the miRNA regulatory system. *DICER1*, another critical gene for miRNA biogenesis, has also been potentially implicated in schizophrenia by a *de novo* CNV associated with illness (Xu et al., 2008). Multiple suggestive level associations with schizophrenia have also been reported with SNPs in miRNA pathway genes (Zhang et al., 2012). Numerous post-mortem studies of miRNA expression in human brain have reported alterations in tissue from individuals with schizophrenia (reviewed in Beveridge and Cairns, 2012). Of particular interest, Moreau et al. (2011) reported a significant overlap between the miRNAs dysregulated in human postmortem tissue from individual with psychotic illness and the miRNAs previously reported dysregulated in brain tissue from a mouse model of 22q11.2DS (Stark et al., 2008). These finding support the involvement of miRNAs in schizophrenia via multiple mechanisms and suggest that there may be overlap in the specific miRNAs involved in 22q11.2DS and non-22q11.2DS mediated cases of schizophrenia.

## The puzzle

Despite the strikingly increased risk for schizophrenia in 22q11.2DS, approximately 75% of individuals with a 22q11.2 deletion do not develop schizophrenia. Other high-risk features of this syndrome, e.g., severe congenital cardiac defects, show comparable levels of reduced penetrance. A number of possible explanations for this variability exist, with these factors potentially acting alone or in combination.

### Chance variation

Stochastic processes can results in individuals with identical genetic endowments and apparently identical environmental exposures developing along different pathways. One could imagine some key cellular factor with a threshold effect on development that is hovering right at the critical level in individuals with 22q11.2DS. Some individuals will be fortunate enough to have just enough of the factor to proceed down the usual developmental pathway, while others may fall just short due to random fluctuations in expression and proceed down an alternative pathway.

### Environmental factors

Triggering or protective environmental factors can interact with identical genomes to produce divergent outcomes in different individuals. Even the intrauterine environment of twins differs, as evidenced by frequently discordant *in utero* HIV transmission (Biggar et al., 2003). The potential divergence of environmental exposures for an adult onset disorder such as schizophrenia would be expected to be even greater.

### Variations in deletion size

The 22q11.2 region contains a set of low copy number repeats that can mediate a variety of specific deletion events. Deletions mediated by different specific LCRs will result in different sets of genes being deleted. Even deletions that appear grossly the same in size may exhibit some variability at the sites of the non-allelic homologous recombination events. These small differences near the deletion break-points may influence the expression of genes critical for determining some of the phenotypic variability of the syndrome. Although there is no evidence to date for this mechanism, careful sequencing studies of the deletion region may shed further light on this possibility.

### Variations in the hemizygous region of 22q11.2

Individuals with 22q11.2DS have only a single remaining copy of the genes in the deletion region. Any susceptibility variants present in these genes no longer have the chance of being compensated for by a non-susceptibility variant on the homolog. A variant in *PIK4CA* has been associated with risk of developing schizophrenia in individuals with 22q11.2DS, but alone is not sufficient to explain the variable penetrance of schizophrenia in these individuals (Vorstman et al., 2009). Sequencing studies of the deletion region will also help clarify this possibility. Interestingly, sequencing of a major congenital cardiac disease gene from the deletion region, *TBX1*, did not reveal such variants for expression of tetralogy of Fallot in 22q11.2DS (Guo et al., 2011).

### Variations elsewhere in the genome

A final potential source of phenotypic variability in 22q11.2DS is genetic variation elsewhere in the genome. Such variants may be considered genetic background effects. The importance of genetic background on variable expressivity and penetrance is well known from the mouse genetics literature, where the same mutation can manifest very differently in different strains (Linder, 2006). This background effect could be as simple as a single modifier locus that interacts with the deletion to determine penetrance of schizophrenia, or it could be similar to the complicated genetic risk proposed for non-22q11.2DS mediated schizophrenia, with the potential for multiple distributed liability loci acting in concert to cause illness. The profoundly increased risk of schizophrenia in 22q11.2DS does suggest that the non-22q11.2 genetic component for these cases of schizophrenia should be simpler than non-deletion mediated forms of the illness, making this an ideal subset for intensive study. Unfortunately, the relatively rarity of 22q11.2 deletions among individuals with schizophrenia (approximately 1% of cases of a disease that affects approximately 1% of the population) has hampered the assembly of the large cohorts necessary to conduct well-powered traditional genome-wide studies.

## A two-hit model

**Figure 2:**
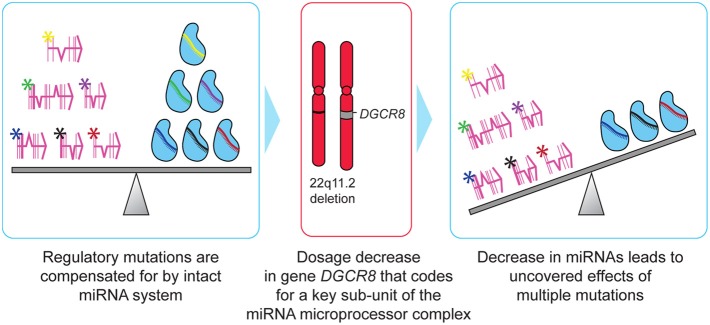
**Two-hit model of schizophrenia in 22q11.2DS.** Mutations that are compensated for by an intact miRNA regulatory system can silently accumulate within individuals, with little selective pressure acting to remove them from the population. Deletion of 22q11.2 results in haploinsufficiency of *DCGR8*, a key gene involved in miRNA biogenesis. The resulting decrease in levels of certain miRNAs results in multiple regulatory mutations simultaneously exerting deleterious effects on the individual, resulting in schizophrenia or other serious abnormalities characteristic of 22q11.2DS.

Canalization is a design principle wherein developmental pathways are stabilized to increase phenotypic reproducibility. This helps the organism withstand genetic or environmental perturbations by providing a buffering system that confers robustness to developmental genetic programs. Hornstein and Shomron have proposed miRNAs as a mechanism for canalization (Hornstein and Shomron, 2006). In this model, miRNA regulation can compensate for variability in gene expression and promote constant levels of protein. Canalized traits have an increased capacity to absorb mutational variance. Specifically, there is a decreased penalty for mutations that alter gene regulation. Altered expression of transcription factors or sequence variation in *cis* binding sites have minimal phenotypic consequences as the miRNA regulatory system compensates for these changes, keeping gene expression at the required level. Because selective pressure is reduced, such mutations may silently accumulate, leading to a state where large numbers of cryptic variations may be distributed throughout the population. A sudden perturbation of the miRNA regulatory system, caused by haploinsufficiency of a key gene in miRNA biogenesis (*DGCR8*), could lead to multiple previously silenced mutations suddenly having an impact on the organism (Figure 2). The expected underlying population heterogeneity in these accumulated regulatory mutations could manifest as a high degree of variability in the specific phenotypic profile seen in individuals with *DGCR8* haploinsufficiency.

DGCR8 is an essential component of the canonical miRNA biogenesis pathway. A non-canonical processing pathway also exits (Babiarz et al., 2008), with mouse knockout studies suggesting that the brain is particularly enriched for these non-canonical miRNAs as compared to other tissues (Babiarz et al., 2011). A mouse model of *DGCR8* haploinsufficiency has also demonstrated that not all miRNAs are equally affected by reduced *DGCR8* levels; despite reductions of *DGCR8* transcript abundance to approximately 60% of wild type levels, only 22% of mature miRNAs assayed exhibited significantly decreased levels in either prefrontal cortex or hippocampus in the haploinsufficient mice (Stark et al., 2008).

## A testable hypothesis

If loss of miRNA silencing of multiple regulatory mutations contributes to the risk of developing schizophrenia in 22q11.2DS, then one would expect these mutation to occur more frequently in genes regulated by the miRNAs most impacted by *DGCR8* haploinsufficiency. Even if tested in a relatively small (by genome-wide association study standards) sample, one would expect to see a pattern of enrichment of association findings to SNPs in or near genes regulated by the 22q11.2DS altered miRNAs. We sought to test this hypothesis using data from a set of 95 families with a 22q11.2DS proband.

## Experiment I—genome-wide association study of schizophrenia in 22q11.2DS

The first experiment in testing our hypothesis was to conduct a GWAS of schizophrenia in 22q11.2DS. To protect against the effects of population stratification, we chose a family based association design. Adults with 22q11.2DS, followed at the Clinical Genetics Research Program, Centre for Addiction and Mental Health (CAMH) at the University of Toronto, Canada, and their families, were studied. Participants provided written informed consent, and the study was approved by the Research Ethics Boards of the University of Toronto, CAMH, and University Health Network. Three hundred and seventeen subjects were included in the study. Comprehensive medical, psychiatric, and family history data were available for all subjects. Experienced psychiatrists provided lifetime DSM-IV psychiatric diagnoses using standard methods, as previously described (Bassett et al., 2007). These individuals comprised 95 families, each with an adult proband confirmed to have a chromosome 22q11.2 deletion by standard methods using metaphase chromosomes from peripheral blood lymphocytes and FISH techniques using a probe, most commonly TUPLE1 (Vysis) or N25 (ONCOR), from the commonly deleted 22q11.2 region (Driscoll et al., 1993). Forty-eight of the 22q11DS probands (26 female and 22 male) were diagnosed with schizophrenia or schizoaffective disorder, while psychotic illness was excluded in the remaining 47 22q11DS probands (28 female and 19 male). Of the 222 additional family members, five (four parents and a half-sibling) also had 22q11.2DS but no psychotic illness; four (two parents and two siblings) were also diagnosed with schizophrenia but did not have 22q11.2DS.

All subjects with available high-quality DNA samples (*n* = 244) were genotyped on Affymetrix 250K Nsp SNP arrays at The Centre for Applied Genomics at the University of Toronto. Genotypes for all CEL files were processed as a single batch using Affymetrix APT software, version 1.10.0. All samples used for further analysis had <5% missing data (*n* = 238). All persons within any pedigree with a detected Mendel error were set to zero for the marker with the error. SNPs were further excluded from all subjects for >2% missingness or deviations from Hardy–Weinberg equilibrium that were significant at *p* < 0.0001. After cleaning, 221,996 SNPs remained for analysis.

Association analysis was conducted using the software package KELVIN 2.4.0, which implements the posterior probability of linkage (PPL) class of models for measuring the strength of genetic evidence (Vieland et al., 2011b). The PPL was initially developed as a linkage analysis method (PPL originally stood for posterior probability of linkage, but now is used to refer to the class of related measures), based upon an acting single-locus likelihood allowing for within-sample heterogeneity. All parameters of the trait model are integrated out of the PPL using simple model averaging, allowing for testing of dominant, recessive, and additive models without the inflationary effects of multiple testing or parameter maximization. The analysis framework is quite flexible, and many additional extensions have been implemented such as analysis of linkage disequilibrium, quantitative traits, liability classes, imprinting, sex specific recombination rates, and epistasis (Bartlett and Vieland, 2007; Huang et al., 2007; Huang and Vieland, 2010; Vieland et al., 2011a). Importantly, the PPL accumulates evidence against linkage or association as well as in favor of it. The PPL is on the probability scale, making its interpretation straightforward. As a measure of statistical evidence, and not a decision-making procedure, there are no associated “significance levels” and it is not interpreted in terms of associated error probabilities nor corrected for multiple testing (Royall, 1997; Vieland and Hodge, 1998; Vieland, 2006).

We chose this analysis approach for two important reasons. First, our study design is complex and we required an analysis approach that could appropriately incorporate all available data into the analysis. While our study fundamentally has a family based association design, it also has elements of a case-control design, with half of the 22q11.2DS probands affected with schizophrenia and half not. We also needed to be able to appropriately model two different clinical elements of interest, 22q11.2 deletion status and schizophrenia status, both of which are present in some of the first degree relatives. Our hypothesis is that mutations segregating within these pedigrees have a greater impact on causing disease in the individuals with 22q11.2 deletions. This led us to model schizophrenia as the primary categorical phenotype of interest for association analysis and model 22q11.2 deletion status as a liability class. In this type of model, individuals with the deletion would be expected to have higher penetrance of schizophrenia with a given risk allele at a susceptibility locus when compared to relatives without the deletion but carrying the same risk allele. KELVIN's flexibility allowed our entire sample to be analyzed simultaneously, incorporating 22q11.2 deletion status as a liability class while testing for association to schizophrenia.

The second important reason for using the PPL framework for our analysis was the desire to use a method that would disambiguate strength of evidence for or against association from low power due to the relatively small size of our sample. The PPL provides a measure of strength of evidence for or against association. Beginning with a given prior probability of association, KELVIN calculates the posterior probability of association based on the provided sample data. If the sample is providing evidence against association, the posterior probability will be lower than the prior probability. If the sample is giving evidence supporting association, the posterior probability will be higher than the prior. The magnitude of the change, up or down, can further be used to determine the strength of the evidence and rank order findings in terms of likelihood of association.

The Affymetrix array data were first used to confirm that all subjects with 22q11.2DS had deletions that included *DGCR8*. Next, the 221,996 SNPs passing our quality control were tested for association. Of these, 9191 were uninformative. Of the remaining SNPs, 198,100 (93%) gave evidence against association, while only 14,705 (7%) provided any evidence for association. Given the small size of the sample (95 small pedigrees), we did not expect to detect compelling evidence for association. Starting from a prior probability of 0.04%, the maximum posterior probability of association was 1.7%, a large increase from the prior but still within the range of what might be seen by chance in a full GWAS. Despite the small magnitude of evidence for association in the best supported SNPs and the expectation that some of these findings were due to chance allele segregations, we reasoned that these top SNPs should be enriched for real association signals. We tested this possibility by a further application of our hypothesis of miRNA mediated risk for schizophrenia in 22q11.2DS.

## Experiment II—test for miRNA target enrichment in 22q11.2DS schizophrenia

The second experiment sought to correlate our GWAS results with miRNA data from two experimental sources. The work of Stark et al. had demonstrated that the levels of some, but not all, miRNAs are significantly decreased in the brains of mice haploinsufficient for *DGCR8* (Stark et al., 2008). Since a list of miRNAs decreased in the brains of humans with 22q11.2DS is not currently available, we began with the list from the mouse model as the best available data, recognizing that noise would undoubtedly be introduced into our analysis by this cross-species approach. The 65 mouse miRNAs reported as downregulated in either prefrontal cortex or hippocampus (Stark et al., 2008) were mapped to their human orthologs and used to query starBase v2.1 (Yang et al., 2011) to determine a list of human genes implicated by two or more CLIP-seq experiments to interact with the miRNAs of interest (Hafner et al., 2010). Overall, 2860 genes were identified as potentially interacting with one or more of the miRNA of interest. Of these, 1047 were predicted to interact with a single miRNA with the remaining genes predicted to interact with between 2 and 34 of the miRNAs investigated (Table 1).

**Table 1:** **Distribution of CLIP-seq interactions with miRNAs of interest**.

**Number of interacting miRNAs**	**Number of genes**
1	1047
2	689
3	395
4	235
5	169
6–10	281
>10	44
Total	2860

Before we could compare this list of genes to our GWAS results, we needed to establish a method for identifying genes that might be regulated by an associated SNP. While c*is*-acting regulatory sequences can act on genes located over 1 Mb away, the sequences that act over the greatest distances tend to be found in gene-poor regions (reviewed in Kleinjan and Coutinho, 2009), suggesting that the physical extent of a candidate region surrounding an LD signal can be modulated by the gene density of the area. We therefore developed an algorithm to map SNPs to potential gene targets based on local gene density. The genomic locations of all RefSeq genes mapped to NCBI Build 36.1 of the human genome were downloaded from the UCSC genome browser (Lander et al., 2001; Kent et al., 2002; Fujita et al., 2011). We then used a four-tiered size approach for searching for potential gene targets. We began by identifying all genes within 25 Kb of a SNP of interest. If no genes were identified, the search window was expanded to 250 Kb on either side of the SNP. If no genes were identified at that step, the window was next expanded to 500 Kb and finally to 1 Mb on either side of the gene. This strategy allows for identification of distant genes from gene-poor regions while limiting the number of genes identified from gene-dense regions. It is important to note, however, that regardless of the interval used, genes are selected purely on the basis of distance from the SNP of interest and all genes within the given window will be identified as potentially regulated by this SNP. This is a very simplistic mapping procedure and even in cases where a SNP of interest actually does have a regulatory function, our algorithm is expected to identify the actual target of that regulatory SNP along with other nearby, but not necessarily regulated, genes. So only some subset of the genes on this list is expected to be of actual interest.

From the 14,705 SNPs producing some positive evidence of association in the GWAS, we arbitrarily selected the 0.5% (73 SNPs) giving the strongest evidence for further analysis. Using the algorithm described above, this set of SNPs identified a set of 72 genes as potential regulatory targets based on location. Of those 72 genes, 12 (17%) had been identified in starBase as potential targets of two or more of the miRNAs observed to be decreased in the *DGCR8* haploinsufficient mice. To determine if this represented enrichment over what would be expected by chance, we conducted a simulation study.

**Figure 3:**
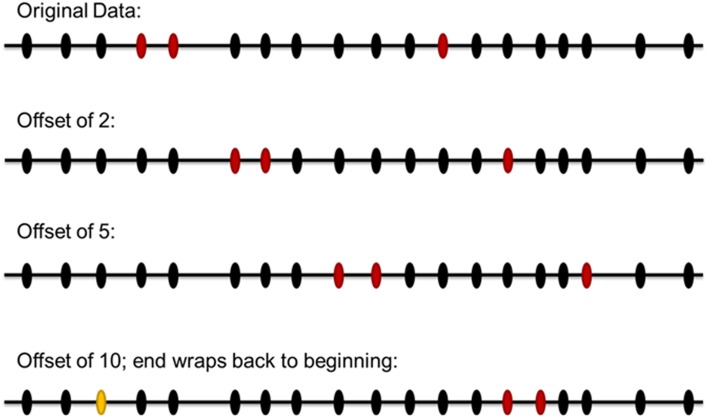
**Scheme for simulation to evaluate significance of experimental results.** The pattern of the positive SNPs from the original data among the total SNPs passing our quality control was defined. For each simulated dataset, this pattern was offset by a random number between 1 and number of SNPs in the original data. In this illustration of the simulation scheme, 19 SNPs are modeled, with the red ovals indicating SNPs of interest. The top line represents the original data. The next two lines represent simulated datasets with offsets of two and five SNPs, respectively. The last line represents a simulated dataset with an offset of ten; in this case the rightmost positive SNPs would be moved off the right end of the set, so it is instead wrapped around to the start of the set, at the position indicated by the yellow oval. For the genome-wide simulation, all chromosomes were laid end-to-end in numerical order to create one continuous array of 221,996 SNPs.

The distribution of the Affymetrix 250K SNPs across genes and the genome is neither perfectly uniform nor random. Similarly, the top association signals are not randomly distributed across the Affymetrix SNPs, with some grouping of positive signals among SNPs in strong linkage disequilibrium with each other. To try to best maintain any effects due to the actual location of the Affymetrix SNPs in the genome and the relative clustering of association signals within nearby SNPs, we devised the following simulation scheme. The pattern of the 73 positive SNPs from the analysis of our real data among the total 221,996 SNPs passing our quality control was defined. For each simulated dataset, this pattern was offset by a random number between 1 and 221,966 (Figure 3). This produced a simulated dataset with the same degree of clustering of SNPs as the real data, but with a different specific set of 73 Affymetrix 250K SNPs selected as targets. These SNPs would then be mapped to genes following the same algorithm that was used for the real data.

Ten thousand simulated datasets were generated by this procedure. The mean number of genes identified by our mapping algorithm in the simulated datasets was 73.0 (s.d. = 11.8), compared to 72 in our real data, suggesting that the simulation procedure successfully generated comparably sized sets of genes. However, among the simulated datasets, only an average of 9.3% of the genes were identified in starBase as potential targets of two or more of the miRNAs observed to be decreased in the *DGCR8* haploinsufficient mice, compared to the 17% observed in the real data set. Based on the distribution of results seen in our simulated data sets, the results observed in the real data set represent a substantial enrichment of genes predicted to be regulated by the miRNAs most disrupted by *DGCR8* haploinsufficiency, significant at *p* = 0.03. This is the result that was predicted by our hypothesis.

## Limitations of this study

The results of these preliminary experiments support our model that the increased risk of schizophrenia in individuals with 22q11.2DS is at least partially due to a loss of miRNA compensation for SNP variants that alter regulation of susceptibility genes. As predicted by our model, our experimental results produced statistically significant enrichment in genes likely to be regulated by the miRNAs decreased by *DCGR8* haploinsufficiency over what would be expected by chance. This result is particularly striking given the number of significant limitations of this study.

First, we used data from *DGCR8* haploinsufficient mice (Stark et al., 2008) as the source of the list of the most disrupted miRNAs. It is possible that the levels of the orthologous miRNAs will not all be similarly altered in humans with 22q11.2DS. Furthermore, the effects of 22q11.2DS on primate and human specific miRNAs will not be informed by the murine model. Therefore, our starting list of miRNAs of interest is incomplete at best but may also contain miRNAs that are not significantly decreased in humans with 22q11.2DS. Ideally, we would start with a list of altered miRNAs obtained through study of human brain tissue from subjects with 22q11.2DS. Given the relative rarity of this syndrome and the general challenges in obtaining human post-mortem brain tissue suitable for gene expression studies, a sizable collection of such tissues will likely be difficult to obtain. Perhaps more logistically feasible would be to use neural tissue derived from induced pluripotent stem cells (iPSCs) made from individuals with 22q11.2DS, although such a derived cell population might include experimentally induced genetic or epigenetic changes. Even profiling of blood samples from individuals with 22q11.2 DS might provide some valuable insights into alterations of primate and human specific miRNAs. Our studies clearly need to be repeated with a list of miRNAs experimentally derived from human source tissue.

Second, we chose to use CLIP-seq data, rather than bioinformatic prediction, to identify potential targets of the miRNAs of interest. CLIP-seq data have the advantage of identifying miRNA targets with better specificity than bioinformatic approaches (Chi et al., 2009; Zisoulis et al., 2010). For example, querying the miRWalk database (Dweep et al., 2011) for predicted targets of the miRNAs of interest using the default setting (target identification by miRanda, miRDB, miRWalk, RNA22, and TargetScan) identifies 10,037 genes predicted to be targets of one or more of the miRNAs of interest, 3.5 times as many as were identified by the available CLIP-seq data. This larger dataset appears to have a worse signal-to-noise ratio than the CLIP-seq data, as repeating our experiments using this very large set of potential target genes failed to produce significant results. More stringent bioinformatic prediction procedures might well perform better. However, the use of CLIP-seq data also has limitations. As an experimentally based approach, the discovered interactions will be limited by the gene expression profiles of the cells that are interrogated. The currently available human CLIP-seq data are still somewhat limited, but as more tissue types are investigated the comprehensiveness of the interactions catalogued will increase and be of even greater value.

Third, we used a sample for this study that is too small to be likely to be able to produce compelling evidence in support of association in the context of a GWAS. While the PPL analysis framework has been demonstrated to have a superior signal-to-noise ratio when compared to other common analysis methods (Huang and Vieland, 2001; Vieland et al., 2001; Logue et al., 2005), the low score threshold for the SNPs investigated in this study clearly overlaps the range of scores that could be seen by chance in a GWAS. Therefore, we expect that some of the SNPs of interest represent false positives. As a group, however, we expect the SNPs of interest to be enriched for true association signals when compared to comparable random sets of SNPs. While subjects with 22q11.2DS are relatively rare, the importance of this syndrome as a model for studying the neurodevelopmental trajectory of schizophrenia has been recognized (Insel, 2010), leading to a new international focus on recruiting such individuals for genetic studies. Larger cohorts will have great power to separate real signals from background noise.

Fourth, we implemented a very simple SNP-to-gene mapping algorithm that identified multiple genes surrounding each associated SNP based solely on physical position. While our procedure used variable size intervals to limit the number of genes identified from the more gene-dense regions of the genome, our algorithm will typically return sets of genes for each SNP of interest, with only a fraction of the identified genes expected to be the target of a SNP with regulatory function. Additional layers of bioinformatic analysis could be added to attempt to further predict the potential regulatory function of the SNPs of interest (or SNPs in strong linkage disequilibrium with them). Functional validation studies of putative regulatory SNPs could also be of utility. Another resource for potentially identifying the regulatory targets of SNPs of interest would be gene expression profiles from individuals with 22q11.2DS. Our hypothesis is that deleterious regulatory mutations are uncovered by damage to the miRNA regulatory system. Therefore, we would expect to see increased expression of the gene targets of the decreased miRNAs. Expression data could be used to further refine the list of putative targets of SNPs of interest.

Fifth, we conducted these experiments using relatively low-density SNP data from Affymetrix 250K NSP arrays. It is quite likely that important signals were missed due to the low SNP density. While this initial study has produced intriguing results, additional studies using a higher density SNP array will be necessary to more comprehensively search the genome for association signals.

## Additional considerations

While we have focused this study on the presence or absence of schizophrenia in individuals with 22q11.2DS, this model could also explain the variability in multiple symptoms of this syndrome. Under the canalization model of Hornstein and Shomron (2006), multiple regulatory mutations can accumulate in the population, free from selective pressure, in the presence of an intact miRNA regulatory system. One would expect the specific distribution of compensated mutations to differ from individual to individual, due to chance segregation of the various deleterious alleles in the population. The striking phenotypic variability of individuals with 22q11.2DS could be due, in part, to the underlying heterogeneity of regulatory mutations that are suddenly uncovered by *DGCR8* haploinsufficiency. One could repeat our experiments with additional phenotypes, such as cardiac malformations, to further test this hypothesis.

Identification and validation of the specific gene targets leading to schizophrenia in the setting of altered miRNAs in 22q11.2DS could provide valuable new insights into this disorder. These genes could represent novel targets for pharmacologic intervention, and it is possible that targeting one or a small number of them could be therapeutically effective. Alternatively, it might be possible to pharmacologically manipulate the miRNA regulatory system more globally. For example, the pri-miRNA processing activity of DGCR8 has been linked to levels of ferric heme, leading to the suggestion that manipulation of the intracellular environment with heme derivatives might have utility in increasing DGCR8 activity (Barr et al., 2012). Such a therapy would directly address the molecular lesion caused by *DGCR8* haploinsufficiency.

While 22q11.2DS contributes to a minority of all cases of schizophrenia, there may well be mechanistic overlap with non-22q11.2DS cases of illness. Post-mortem analysis of human brain tissue has indicated a significant overlap in the miRNAs dysregulated in non-22q11.2DS individuals with psychotic illness and those depleted in a mouse model of 22q11.2DS (Stark et al., 2008; Moreau et al., 2011), suggesting the potential for shared mechanisms between etiologically different forms of schizophrenia. Further understanding of 22q11.2DS may well inform our global understanding of the causes, and potential treatment, of all schizophrenia.

### Conflict of interest statement

Dr. Brzustowicz serves as a consultant for the Janssen Pharmaceutical Companies of Johnson & Johnson. Dr. Bassett declares that the research was conducted in the absence of any commercial or financial relationships that could be construed as a potential conflict of interest.
